# Inhibin βE (*INHBE*) is a possible insulin resistance-associated hepatokine identified by comprehensive gene expression analysis in human liver biopsy samples

**DOI:** 10.1371/journal.pone.0194798

**Published:** 2018-03-29

**Authors:** Masakazu Sugiyama, Akihiro Kikuchi, Hirofumi Misu, Hirobumi Igawa, Motooki Ashihara, Youichi Kushima, Kiyofumi Honda, Yoshiyuki Suzuki, Yoshiki Kawabe, Shuichi Kaneko, Toshinari Takamura

**Affiliations:** 1 Research Division, Chugai Pharmaceutical Co., Ltd., Gotemba, Shizuoka, Japan; 2 Department of Endocrinology and Metabolism, Kanazawa University Graduate School of Medical Sciences, Kanazawa, Ishikawa, Japan; 3 Department of System Biology, Kanazawa University Graduate School of Medical Sciences, Kanazawa, Ishikawa, Japan; 4 PRESTO, Japan Science and Technology Agency, Kawaguchi, Saitama, Japan; INRA, FRANCE

## Abstract

The liver plays a major role in whole-body energy homeostasis by releasing secretory factors, termed hepatokines. To identify novel target genes associated with insulin resistance, we performed a comprehensive analysis of gene expression profiles using a DNA chip method in liver biopsy samples from humans with varying degrees of insulin resistance. Inhibin βE (*INHBE*) was identified as a novel putative hepatokine with hepatic gene expression that positively correlated with insulin resistance and body mass index in humans. Quantitative real time-PCR analysis also showed an increase in *INHBE* gene expression in independent liver samples from insulin-resistant human subjects. Additionally, *Inhbe* gene expression increased in the livers of db/db mice, a rodent model of type 2 diabetes. To preliminarily screen the role of *Inhbe in vivo* in whole-body energy metabolic status, hepatic mRNA was knocked down with siRNA for *Inhbe* (siINHBE) in db/db mice. Treatment with siINHBE suppressed body weight gain during the two-week experimental period, which was attributable to diminished fat rather than lean mass. Additionally, treatment with siINHBE decreased the respiratory quotient and increased plasma total ketone bodies compared with treatment with non-targeting siRNA, both of which suggest enhanced whole-body fat utilization. Our study suggests that *INHBE* functions as a possible hepatokine to alter the whole-body metabolic status under obese insulin-resistant conditions.

## Introduction

Type 2 diabetes (T2D) is characterized by progressive decline in insulin secretion because of pancreatic β-cell dysfunction and by insulin resistance in the liver and peripheral tissues, such as skeletal muscle and adipose tissue [1–3]. In an insulin-resistant state, insulin action is impaired. This promotes hepatic glucose production and reduces peripheral glucose uptake, inducing compensatory hyperinsulinemia. Consequently, the pancreas cannot keep up with insulin demand, and eventually pancreatic β-cell dysfunction occurs, which leads to the development of T2D. The molecular mechanisms underlying insulin resistance have been determined, but its details have not been fully clarified.

An approach using transcriptomes of liver tissues from patients with T2D is expected to resolve the molecular mechanisms associated with hepatic insulin resistance and identify therapeutic targets for T2D [4–9]. A previous study showed that various hepatic genes were differentially expressed in the liver of patients with T2D [4]. Using comprehensive analyses of gene expression profiles in human liver samples, selenoprotein P was identified as a hepatokine that causes insulin resistance and hyperglycemia [10].

The present study aimed to identify novel genes encoding functional target proteins associated with insulin resistance. For this purpose, comprehensive gene expression analysis using a DNA chip method was performed to explore the target genes in liver biopsy samples from insulin-resistant human subjects. Inhibin βE (*INHBE*) was identified as a candidate through these analyses.

*INHBE* is a growth factor that belongs to the transforming growth factor-β (TGF-β) family. *INHBE* mRNA is predominantly expressed in the liver [11–15], and *INHBE* is involved in the regulation of liver cell growth and differentiation [16, 17]. Furthermore, a previous study reported that insulin stimulated *INHBE* expression in liver cells, and *INHBE* mRNA was upregulated in the livers of diet-induced obese mice, suggesting that *INHBE* is implicated in glucose metabolism [18]. However, the association between insulin resistance and *INHBE* expression in the liver of humans has not been determined. Moreover, the role of *INHBE* in metabolic functions has not been clarified in obese insulin-resistant conditions.

Therefore, in addition to the study in humans, to preliminarily analyze the metabolic function of *INHBE*, an *in vivo* small interfering RNA (siRNA) technique was applied to genetically obese insulin-resistant db/db mice. From these investigations, *INHBE* emerged as a possible hepatokine associated unexpectedly with whole-body metabolism under obese insulin-resistant conditions.

## Methods

### Human subjects

Samples for DNA chip analysis were obtained from 15 subjects with varying degrees of insulin resistance and fasting plasma glucose (FPG) levels of less than 126 mg/dL who were admitted to Kanazawa University Hospital. Subjects with type 2 diabetes were treated with diet therapy alone or insulin. Oral hypoglycemic agents were not used for the treatment of any subject. Liver tissue samples were obtained by percutaneous needle biopsy. The subjects were divided into two groups according to their homeostasis model assessment of insulin resistance (HOMA-IR) levels. Subjects with HOMA-IR level < 2.0 were defined as the low HOMA-IR group, while the other subjects were assigned to the high HOMA-IR group. The clinical characteristics of these two groups are listed in Table 1. Subjects whose FPG levels were less than 126 mg/dL were analyzed to compare subjects with pre-diabetes with those with normal insulin sensitivity. This analysis was performed to identify target genes that were more sensitive to insulin resistance than to plasma glucose.

**Table 1:** Clinical characteristics of subjects for DNA chip and qRT-PCR analyses.

	DNA chip analysis						qRT-PCR analysis					
	Low			High			Low			High		
	HOMA-IR			HOMA-IR			HOMA-IR			HOMA-IR		
**No. (male:female)**	6 (1:5)			9 (6:3)			5 (2:3)			5 (4:1)		
**Age (yr)**	58	±	3	40	±	3**	55	±	4	56	±	5
**BMI (kg/m**^**2**^**)**	26.3	±	1.9	26.1	±	1.7	26.5	±	1.6	29.4	±	2.1
**FPG (mg/dL)**	99	±	5	100	±	8	96	±	4	104	±	3
**HbA1c (%)**	5.5	±	0.2	5.6	±	0.3	5.2	±	0.3	6.9	±	0.8
**HOMA-IR**	1.07	±	0.11	3.77	±	0.96*	1.59	±	0.19	3.35	±	0.51*
**AST (IU/L)**	20	±	2	39	±	6*	32	±	10	31	±	6
**ALT (IU/L)**	18	±	3	83	±	24*	40	±	16	40	±	9
**Total cholesterol (mg/dL)**	204	±	20	198	±	18	200	±	22	166	±	17
**Triglyceride (mg/dL)**	128	±	28	136	±	23	110	±	24	139	±	24
**Hepatic *INHBE* mRNA**	1.00	±	0.15	1.66	±	0.17*	1.00	±	0.08	2.10	±	0.36*

BMI: Body mass index, FPG: Fasting plasma glucose, Hb: Hemoglobin, HbA1c indicates glycosylated hemoglobin, AST: aspartate transaminase, ALT: alanine transaminase, HOMA-IR: Homeostasis model assessment of insulin resistance. Data are expressed as means ± SEM.

* *P* < 0.05

** *P* < 0.01, *vs*. low HOMA-IR group.

Additionally, samples for quantitative real-time reverse-transcription PCR (qRT-PCR) analysis were obtained from 10 subjects independent from those used for the DNA chip analysis (Table 1). These subjects were also divided into low or high HOMA-IR groups as described above. With the exception of HOMA-IR level, the clinical characteristics of these two groups were comparable.

All subjects provided written informed consent for this study. The study protocol was approved by the Ethics Committee of Kanazawa University Graduate School of Medical Sciences (Approval No. 184) and Chugai Pharmaceutical Co., Ltd (Approval No. G20) and was performed in accordance with the Declaration of Helsinki.

### DNA chip and qRT-PCR analysis of human liver tissue

Extraction of RNA from human liver tissues, Affymetrix gene chip hybridization, DNA chip analysis, and qRT-PCR analysis were performed as previously described [5, 7, 10]. Briefly, total RNA was extracted with a kit (Micro RNA Isolation Kit; Stratagene, La Jolla, CA, USA) and purified with the RNeasy MiniElute Cleanup Kit (QIAGEN, Hilden, Germany). Total RNA was then labelled according to the small sample labeling protocol recommended by the manufacturer (SuperScript Choice System; Invitrogen Thermo Fisher Scientific, Waltham, MA, USA). The cDNA was amplified by an *in vitro* transcription step with T7 RNA polymerase (MEGAscript T7 Kit; Ambion, Thermo Fisher Scientific). Biotin-labelled ribonucleotides were incorporated in the cRNA using a kit (Bio Array High Yield RNA Transcript Labelling Kit; Enzo Diagnostics, Farmingdale, NY, USA) during RNA amplification. Ten micrograms of labelled and fragmented cRNA was then hybridized onto a Human Genome U133 Plus 2.0 Array (Affymetrix, Santa Clara, CA, USA) for 16 h at 45°C. Post-hybridization staining was conducted according to the manufacturer’s (Affymetrix) instructions. Finally, chips were scanned (GeneChip Scanner3000; Affymetrix). A scanned image was quantified using Expressionist 5.3 (Genedata, Basel, Switzerland).

Double-stranded cDNA was used as a template for qRT-PCR. To do this, we used a sequence detection system (ABI Prism 7700; Applied Biosystems, Foster City, CA, USA). The sets of primers and TaqMan probes used are proprietary to Applied Biosystems (Assays-on-Demand gene expression product). To control for variation in the amount of DNA available for PCR in the different samples, gene expression of the target sequence was normalized relative to expression of an endogenous control, beta-actin RNA (beta-actin RNA TaqMan Control Reagent Kit; Applied Biosystems).

### Isolation of mouse primary hepatocytes and siRNA study

Livers from C57BL/6J mice were digested by collagenase perfused through the portal vein using a two-step collagenase perfusion method. Hepatocytes were collected as pellets by centrifugation at 50 × g for 5 min. Hepatocytes were cultured in DMEM containing 10% FBS on collagen-I coated plates. siRNA studies were performed using the siRNAs specified below. The siRNAs were purchased from Thermo Fisher Scientific (Ambion® In Vivo siRNA; Ambion, Thermo Fisher Scientific) and incorporated chemical modifications for superior serum stability with *in vivo* delivery. Mouse *Inhbe* siRNA (siINHBE) had the following sequence:

5’-CAGCTTTGCTACCATCATA-3’ (sense). Non-targeting siRNA (siNON) was also used, and this siRNA had no significant homology with any known gene in mouse, rat, or human (Ambion® *In Vivo* Negative Control #1 siRNA). The siRNAs were transfected into hepatocytes using Invivofectamine 2.0 transfection reagent (Invitrogen, Thermo Fisher Scientific) 1 day after plating.

### Animals

Male db/db, KK-Ay, and C57BL/6J mice were obtained from CLEA Japan, Inc. (Tokyo, Japan). All animals were housed individually in polycarbonate animal cages with a 12-h light/dark cycle and allowed free access to food and water. Body weight and food intake were evaluated in animals treated with siRNA or with negative control RNA from 6 to 8 weeks of age after first dosing. In the daytime on days 2, 7, and 14 after first dosing of siRNA, mice in a freely fed state were anesthetized, and whole blood was collected via inferior vena cava or heart. The animals were killed by exsanguination under anesthesia, and livers were isolated. The tissues were weighed and stored at 80°C until use. Plasma total ketone bodies of mice in a freely fed state were measured with an automated analyzer (TBA-120FR, Toshiba Medical Systems Co, Tochigi, Japan). Experimental procedures and animal care were performed in accordance with the requirements of the Institutional Animal Care and Use Committee at Chugai Pharmaceutical Co., Ltd. The protocol was approved by the Institutional Animal Care and Use Committee at Chugai Pharmaceutical Co., Ltd.

### siRNA experiment in db/db mice

To transfect siRNA *in vivo*, Invivofectamine 2.0 was used according to the manufacturer’s protocol. For the *in vivo* mouse study, we intravenously injected mouse siINHBE or siNON (7 mg/kg; 10 mL/kg) mixed with the transfection reagent into the tail vein of 6-week-old db/db mice. Administration of siRNA was performed on day 0 (first dosing) and day 4 (second dosing).

### qRT-PCR for mRNA measurements in mouse samples

Total RNA was isolated from primary hepatocytes with an RNeasy 96 kit (QIAGEN) or from frozen liver tissue with TRIzol reagent (Invitrogen, Thermo Fisher Scientific) followed by an RNeasy mini kit (QIAGEN). RT-PCR was performed with a QuantiTect Probe RT-PCR kit (QIAGEN) and probes (TaqMan Gene Expression Assays for mouse, *Inhbe*: Mm03023993_m1, 18s rRNA: Hs99999901_s1; Applied Biosystems). The relative amounts of each RNA in the total RNA were analyzed using a real-time RT-PCR quantitative system (7900HT Fast Real Time PCR system, Applied Biosystems) and calculated with 18s RNA as an internal control.

### Protein isolation and measurement of INHBE protein levels

Liver tissue samples were homogenized using a Polytron homogenizer in RIPA buffer (Merck Millipore, Billerica, MA, USA) containing a Complete® Mini EDTA-free cocktail tablet (Roche Diagnostics, Basel, Switzerland) and PhosSTOP phosphatase inhibitor cocktail tablet (Roche Diagnostics). After sonication with a Bioruptor (Cosmo Bio, Tokyo, Japan), the tissue lysates were further solubilized by continuous stirring for 1 h at 4°C and centrifuged to remove insoluble material. The supernatants (6.6 μg of protein per sample) were used for measurement of hepatic INHBE protein levels. The concentrations of INHBE in the liver and plasma were determined using an ELISA kit for mouse INHBE (Wuhan USCN Business, Houston, TX, USA) according to the manufacturer’s instructions.

### Indirect calorimetric studies in db/db mice

A comprehensive animal metabolic monitoring system (Muromachi Kikai Co., Ltd., Tokyo, Japan) was used to evaluate energy expenditure (EE), respiratory quotient (RQ), and locomotor activity. EE and RQ were calculated from gas exchange data: EE = (3.815 + 1.232 × RQ) × oxygen consumption (VO_2_), with RQ determined as the ratio of carbon dioxide production (VCO_2_) to VO_2_. Mouse activity was measured in both horizontal and vertical directions using infrared beams and counting the beam breaks during a specified period.

### Body composition by microcomputed tomography (microCT)

Mice were anesthetized with isoflurane, and body composition was evaluated with a microCT scanner (eXplore Locus, General Electric Company, Tokyo, Japan) and image analysis software (Micro View, General Electronic Company) [19]. The CT value of olive oil (Wako Pure Chemical Industries Ltd, Osaka, Japan) was used as the standard density for adipose tissue. The CT value of a standard phantom with calibration cells containing calcium hydroxyapatite (Kyoto Kagaku, Kyoto, Japan) at a concentration equivalent to 400 mg cm^-3^ was used as the standard density for bone. Bone volume was evaluated using a voxel with a higher CT value than a value in the standard phantom. Body lean mass was evaluated using a voxel with a CT value between the standard phantom and olive oil.

### Statistical analysis

All data were analyzed using the SAS System for Windows, Release 8.02 (SAS Institute Japan, Tokyo, Japan), JMP 9.0.2 or JMP 13.2.1 (SAS Institute Japan, Tokyo, Japan). Numeric values are expressed as mean ± SEM. Differences between two groups were assessed by unpaired *t*-test if the normal distribution of data was not rejected as *p* ≧ 0.05 using Shapiro-Wilk normality test. Mann-Whitney U test was applied if the normal distribution was rejected using Shapiro-Wilk test.

## Results

### Selection of possible candidates for metabolic phenotype-related genes by comprehensive gene expression analysis in human liver biopsy samples

**Fig 1:**
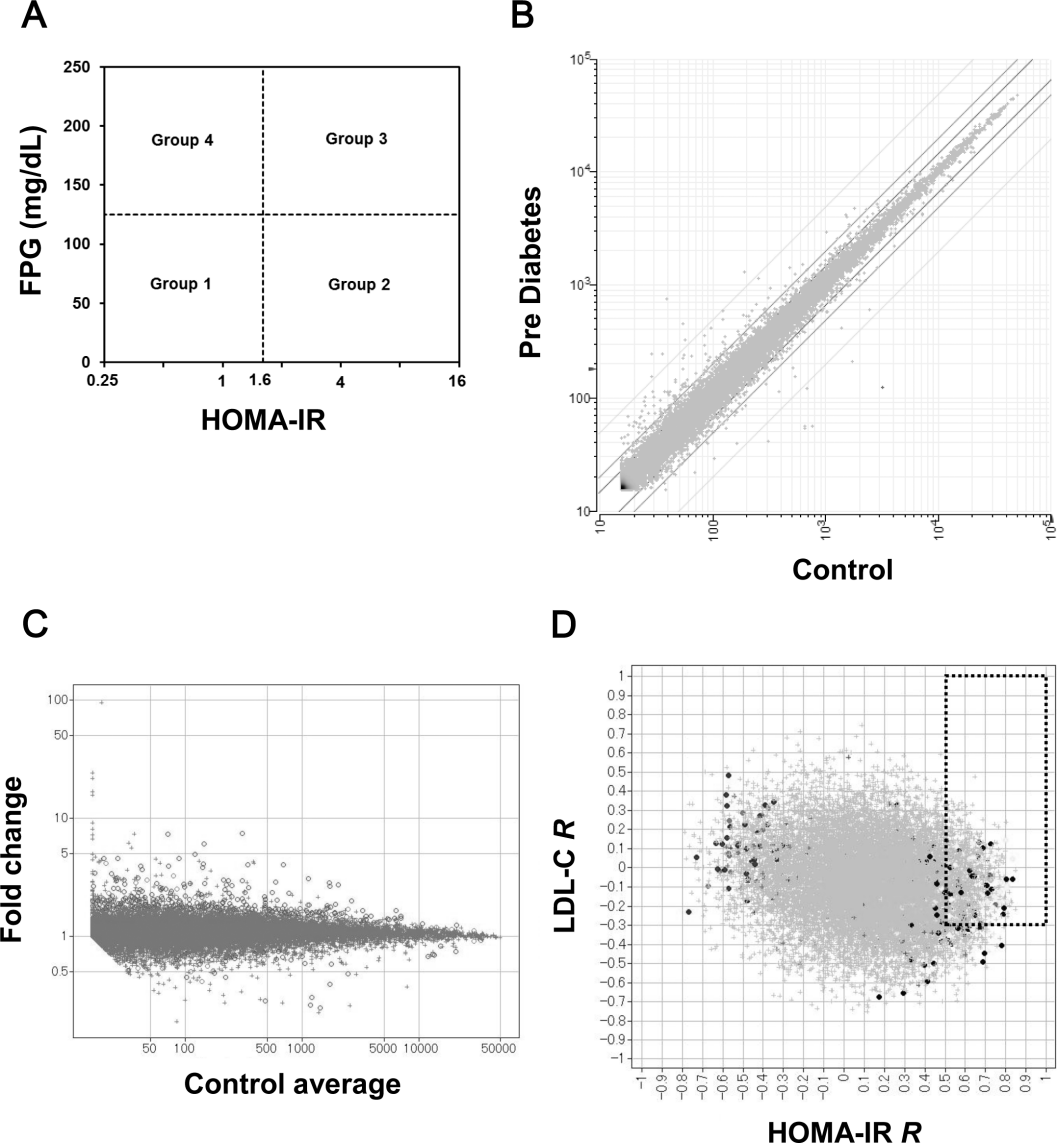
Exploration of novel target genes associated with insulin resistance by comprehensive gene expression analysis in human liver biopsy samples. (A) Human subjects were grouped into four groups based on characteristics of insulin resistance and blood glucose. Subjects with high fasting plasma glucose (FPG) (≥ 125 mg/dL) and high HOMA-IR (≥ 1.6) were assigned to the Diabetes group or Group 3. Subjects with normal FPG and high HOMA-IR were assigned to the Pre-Diabetes group or Group 2. Subjects with low FPG and low HOMA-IR were assigned to the Control group or Group 1. (B) The expression data from approximately 50000 probes set on the DNA chip were quantified. The mean expression level values in Group 1 (control) and Group 2 (pre-diabetes) for each probe were plotted. (C) The fold change of the mean value in Group 2 (n = 9) relative to that in Group 1 (n = 6) for each probe was calculated (Group 2/Group 1 ratio) and plotted against the mean signal in Group 1 for each probe. (D) Spearman’s rank correlation coefficients (*R)* of signals for each probe versus HOMA-IR were calculated. Additionally, the *R* values of signals for each probe versus low density lipoprotein (LDL) cholesterol were plotted against *R* of HOMA-IR. Probes with *R* versus HOMA-IR higher than 0.5 and with *R* of LDL-C higher than −0.3 were selected.

DNA chip analyses in humans were performed to explore novel target molecules that were associated with insulin resistance and whole-body metabolic phenotype. First, human subjects were grouped into four groups based on characteristics of insulin resistance and blood glucose, as shown in Fig 1A. Subjects with low FPG (< 125 mg/dL) and low HOMA-IR (< 1.6) were assigned to the Control group or Group 1. Subjects with low FPG and high HOMA-IR (≥ 1.6) were assigned to the Pre-Diabetes group or Group 2. Subjects with high FPG (≥ 125 mg/dL) and high HOMA-IR were assigned to the Diabetes group or Group 3. Subjects with high FPG and low HOMA-IR were assigned to Group 4. To investigate the mRNA expression associated with insulin resistance, we primarily analyzed subjects in Groups 1 and 2. Data from approximately 50000 probes set on the U133 Plus 2.0 Array were quantified, and the GC Robust Multi-array Average (GCRMA) algorithm was applied to both normalize and summarize the data.

The mean expression levels in Groups 1 and 2 for each probe were plotted, as shown in Fig 1B. The fold change of the mean value in Group 2 (n = 9) relative to that in Group 1 (n = 6) for each probe was calculated (Group 2/Group 1 ratio) and plotted against the mean signal in Group 1 for each probe, as shown in Fig 1C. Probes for a gene without an assigned gene symbol or with an ambiguous annotation were removed from the subsequent analysis. Probes with a Group 2 to Group 1 fold change ratio close to 1 were also removed from further analysis. Probe sets in the Control group were sub-grouped by mean signal intensity as follows: < 20, 20–30, 30–100, 100–200, 200–500, 500–1000, 1000–2000, 2000–3000, 3000–5000, 5000–10000, 10000–20000, and > 20000. The average value of the bottom 2 logarithms of the Group 2/Group 1 ratio for all probes in each sub-group was calculated (for example, $\overset{-}{X}$_100–200_). The absolute values of the differences between *X* (bottom 2 logarithms of the Group 2/Group 1 ratio for each probe) and $\overset{-}{X}$ were calculated against each upregulation or downregulation (for example, |*X*_*probe A*_*−$\overset{-}{X}$*_100–200_|_up_ or |*X*_*probe A*_*−$\overset{-}{X}$*_100–200_|_down_). Standard deviations (SD) of |*X–$\overset{-}{X}$*| were caluculated against each upregulation or downregulation in each sub-group.

Probes with |*X–$\overset{-}{X}$*| greater than 2-fold of their standard deviations were selected as the first hit (approximately 1000 probes). Probes with signals in Group 2 that were significantly higher than those in Group 1 (Student’s *t*-test, *p* < 0.05) were then further selected (approximately 300 probes). Spearman’s rank correlation coefficients (*R*) of the signals for each probe versus HOMA-IR were calculated. *R* values for the signals for each probe versus total cholesterol, LDL cholesterol, HDL cholesterol, triglycerides, histopathological scores for hepatic steatosis, and the mean centroid [4, 5] of expression in the probe set, which was annotated for fat synthesis-related genes (*PKLR*, *HMGCR*, *PPARG*, *GPAM*, *ACLY*, *LDLR*, *ACACA*, *FASN*, *PCSK9*, *SREBF1*, *ME1*, *ME2*, *ME3*, *ELOVL6*, *SCD*, and *ACSL1*), were also calculated. The *R* values for lipid-related parameters were then used to select candidate genes in addition to *R* for HOMA-IR. For example, as indicated in Fig 1D, probes with *R* values higher than 0.5 for HOMA-IR and −0.3 for LDL were selected, because downregulation of the genes corresponding to these probes possibly contributes to decreased HOMA-IR and either unchanged or decreased LDL-C (111 probes). *R* values for total cholesterol, LDL cholesterol, HDL cholesterol, triglycerides, steatosis score, and mean centroid for fat synthesis-related genes were assessed similarly to Fig 1D, and 139 probes were selected. Moreover, probes with Group 3 expression levels that were obviously lower than those in Group 2 were excluded, and approximately 80 probes were selected. Fifteen candidate genes were then selected based on the factors below. Namely, we considered tissue distribution (liver- and metabolism-related tissues, including adipose tissue and skeletal muscle) of mRNA expression, higher *R* for lipid-related parameters (considering the possibility of improving dyslipidemia by downregulation of the genes), druggability for drug discovery, and presence of a previous report in which target genes were investigated in a metabolism-related area (ease of research in the future). The expression of 15 genes in human liver biopsy samples (n = 10), which were different from those analyzed by DNA chip analysis and diabetic obese mouse liver, were analyzed to confirm the validity (data not shown except for *INHBE*). Three genes were selected through these processes. These three genes were subjected to analysis of metabolic phenotype using the *in vivo* siRNA technique in diabetic obese mouse (data not shown except for *INHBE*). *INHBE* was the only gene that impacted the metabolic phenotype *in vivo*.

### Hepatic *INHBE* mRNA expression was elevated with insulin resistance in humans

**Fig 2:**
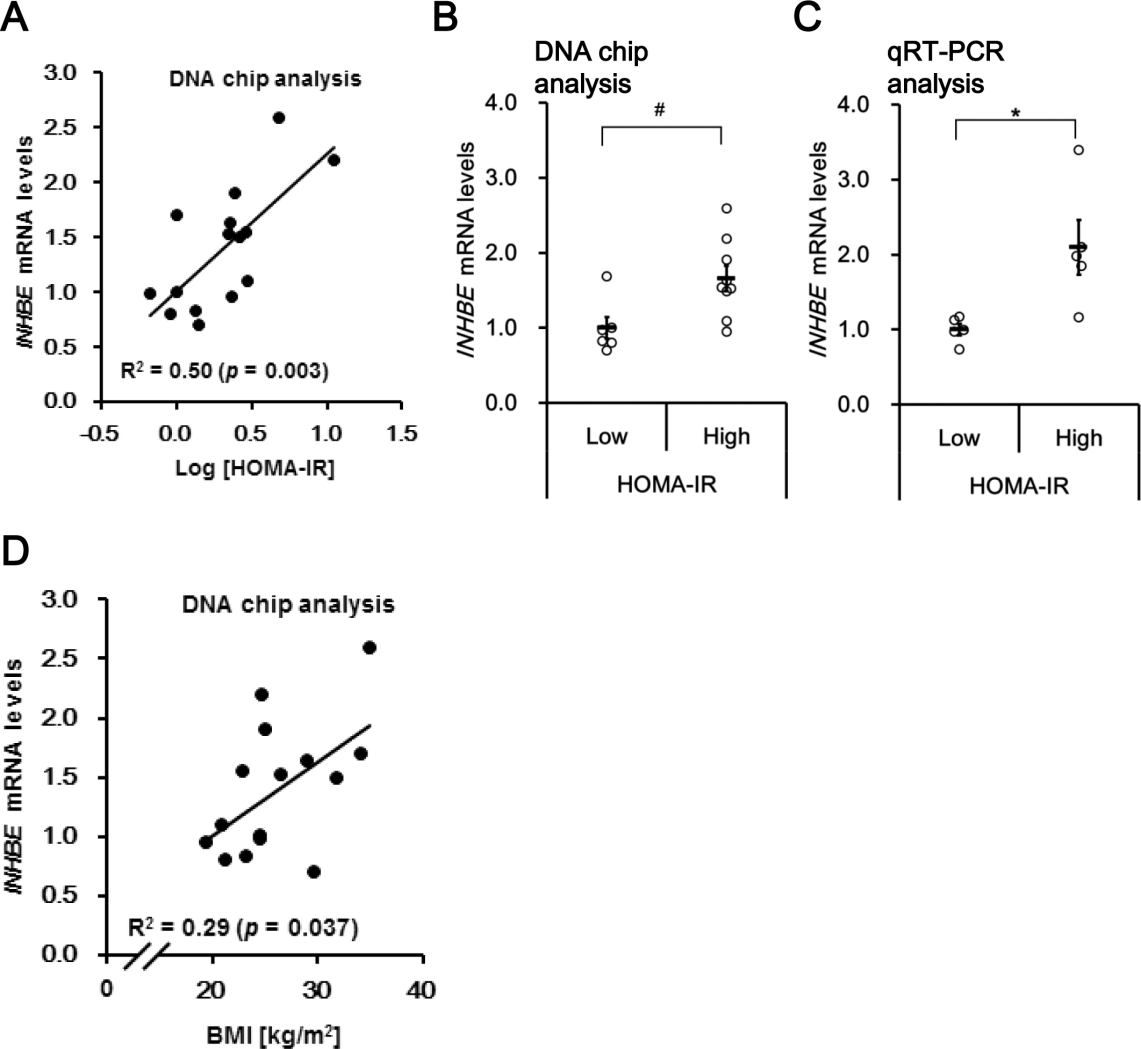
Hepatic *INHBE* mRNA expression was increased in humans with high insulin resistance and body mass index. (A) Correlation between the homeostasis model assessment of insulin resistance (HOMA-IR) level and *INHBE* mRNA expression in the livers of human subjects. *INHBE* mRNA level was quantified by DNA chip. (B) Hepatic *INHBE* mRNA levels (DNA chip) in human subjects in the low (n = 6) and high HOMA-IR groups (n = 9). (C) Hepatic *INHBE* mRNA level by quantitative real-time reverse-transcription PCR (qRT-PCR) in human subjects in the low (n = 5) and high HOMA-IR groups (n = 5). (D) Correlation between body mass index (BMI) and mRNA expression of *INHBE* (DNA chip) in the livers of human subjects. Data in (B) and (C) are expressed as mean ± SEM. Differences between the two groups were assessed by Mann-Whitney U test (B) or unpaired *t*-test (C). ^#^: *P* < 0.05 (B), *: *P* < 0.05 (C).

*INHBE* was identified as a candidate hepatokine from our investigation as described above. Namely, analyses using liver biopsy samples from 15 subjects with varying degrees of insulin resistance indicated a positive correlation between hepatic *INHBE* mRNA and the HOMA-IR level (Fig 2A). Hepatic *INHBE* mRNA level was significantly higher (by a factor of 1.7) in the high HOMA-IR group than in the low HOMA-IR group (Table 1, Fig 2B). Elevation of hepatic *INHBE* mRNA expression in high HOMA-IR subjects was confirmed by qRT-PCR analysis of liver samples from independent subjects (Table 1, Fig 2C). Moreover, there was also a positive correlation between hepatic *INHBE* mRNA level and body mass index (Fig 2D).

### Selection of siRNA for *in vivo* analysis to explore the whole-body metabolic function of *INHBE*

**Fig 3:**
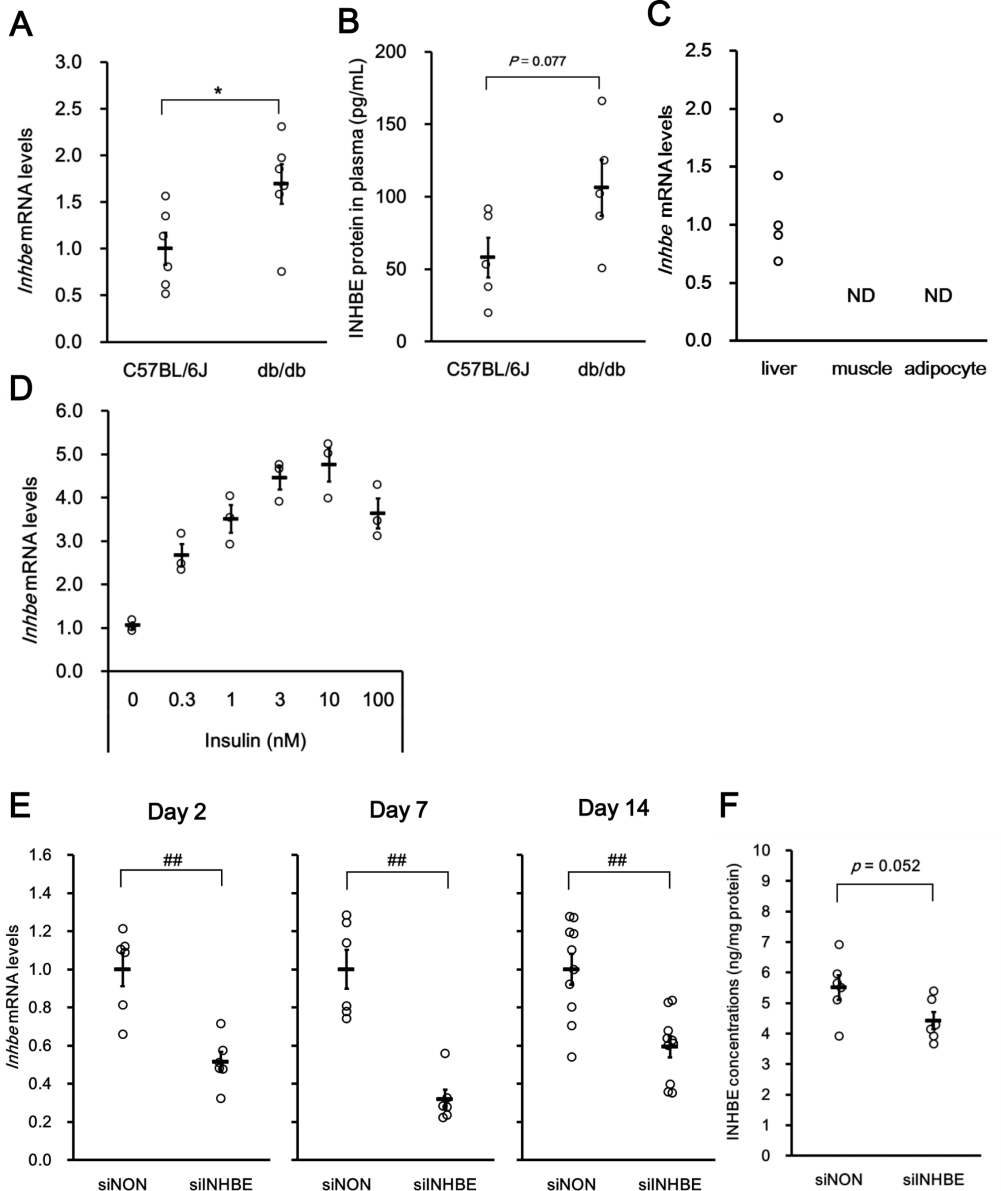
Hepatic *Inhbe* mRNA expression in mice. (A) Hepatic *Inhbe* mRNA levels were assessed by qRT-PCR in control animals (C57BL/6J mice; n = 6) and an animal model of type 2 diabetes at 8–12 weeks of age (db/db mice; n = 6). Animals in a freely fed state were sacrificed in the daytime. (B) INHBE protein levels in plasma were assessed by ELISA in control animals (C57BL/6J mice; n = 5) and an animal model of type 2 diabetes at 8 weeks of age (db/db mice; n = 5) in a freely fed state. (C) *Inhbe* mRNA expression in the liver, skeletal muscles and epididymal adipose tissue of an animal model of type 2 diabetes at 8 weeks of age (db/db mice; n = 5) were assessed by qRT-PCR; ND, not determined. (D) Insulin increased *Inhbe* mRNA expression in mouse primary hepatocytes. Insulin-induced expression of *Inhbe* mRNA was observed in cells treated with culture medium containing 8-(4-chlorophenylthio)-cAMP (8-CPT-cAMP) and glucocorticoid. Data are expressed as mean ± SEM of three wells. (E) Hepatic *Inhbe* mRNA levels in the siNON- and siINHBE-treated groups on days 2 (n = 6), 7 (n = 6), and 14 (n = 10) after the first dosing. (F) Hepatic Inhbe protein levels measured by ELISA in the siNON- (n = 6) and siINHBE-treated groups (n = 6) on day 7 after the first dosing. Data are expressed as mean ± SEM. Differences between the two groups were assessed by unpaired *t*-test (A, E) or Mann-Whitney U test (D). *: *P* < 0.05 (A), ^##^: *P* < 0.01 (D).

Similar to the results in human clinical specimens, 1.7 times higher expression of *Inhbe* mRNA in the liver of db/db mice compared with that in C57BL/6J mice was observed (Fig 3A). Higher trend of Inhbe protein levels in plasma in db/db mice than that in C57BL/6J was also found (Fig 3B). Higher expression of *Inhbe* mRNA was also observed in KK-Ay mice compared with that in C57BL/6J mice (S1 Fig). On the other hand, *Inhbe* mRNA was not detected by using qRT-PCR in the skeletal muscles and epididymal adipose tissue of db/db mouse (Fig 3C). To explore the inducer of *Inhbe* expression associated with insulin resistance, the effect of insulin on *Inhbe* mRNA expression was investigated in mouse primary hepatocytes. Insulin treatment dose-dependently upregulated *Inhbe* mRNA level in mouse primary hepatocytes (Fig 3D) in the presence of 8-(4-chlorophenylthio)-cAMP (8-CPT-cAMP) and glucocorticoid.

To select siRNA for *Inhbe* gene silencing, 13 siRNAs were tested in mouse primary hepatocytes. Only one siRNA knocked down the *Inhbe* gene in mouse primary hepatocytes among the 13 siRNAs analyzed. This siRNA (siINHBE) decreased *Inhbe* mRNA expression by up to more than 80% compared with that of siNON at a concentration of 10 nM (S2 Fig). Next, the knockdown potency of this siRNA *in vivo* was analyzed in obese insulin-resistant db/db mice. Hepatic *Inhbe* mRNA levels were suppressed to 51±5% of that of the negative control on day 2, 32±5% on day 7, and 63±6% on day 14 after the first dosing (Fig 3E). Hepatic INHBE protein levels tended to be suppressed by siINHBE treatment compared with those of siNON treatment on day 7 after the first dosing (Fig 3F). These results suggest that this siRNA was appropriate to explore whole-body metabolic function of *Inhbe in vivo*.

### *Inhbe* gene silencing in the liver suppressed body weight gain and fat volume

**Fig 4:**
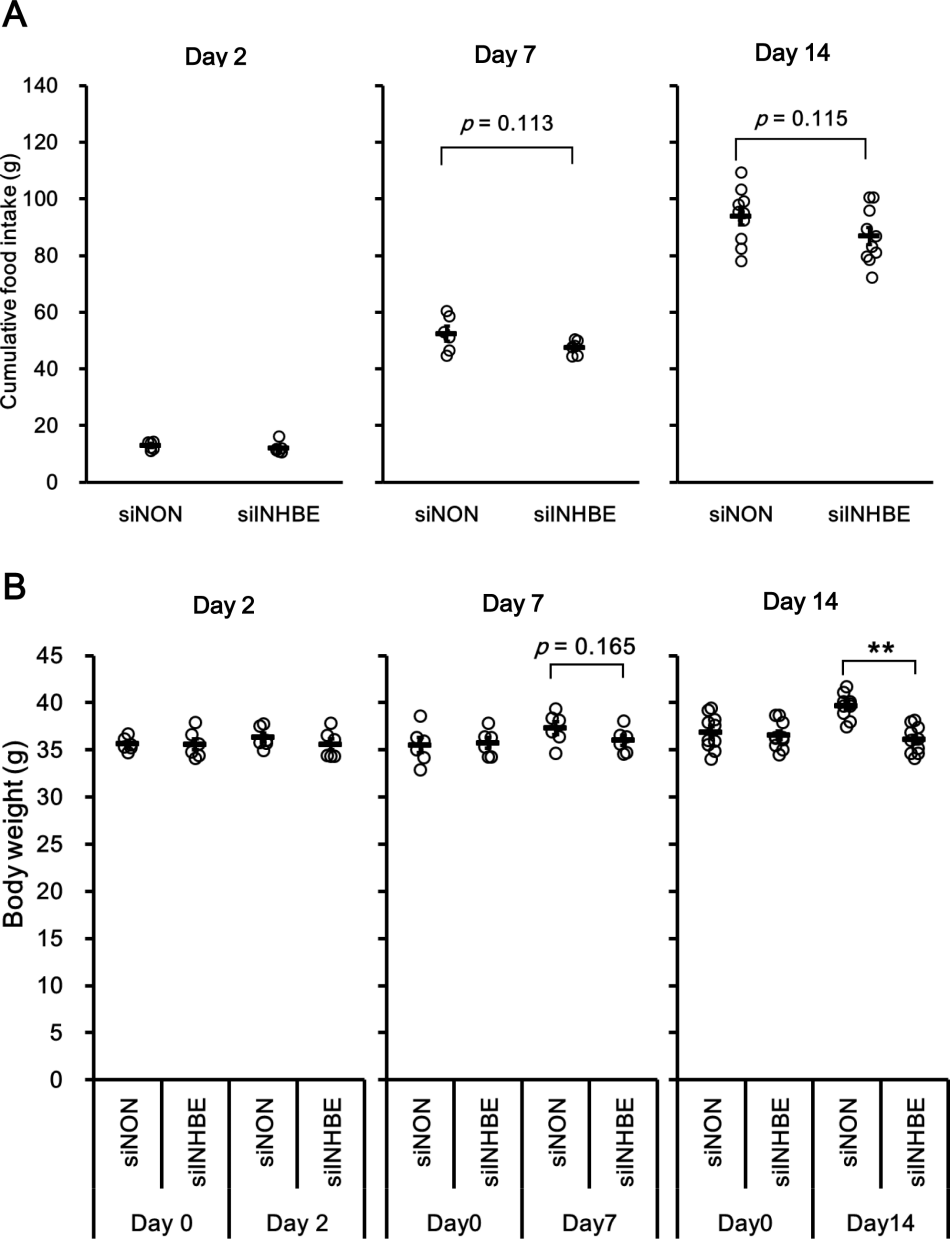
Knock down of hepatic *Inhbe* mRNA in db/db mice suppressed body weight gain. (A) Cumulative food intake in the siNON- and siINHBE-treated groups on days 2 (n = 6 for each group), 7 (n = 6 for each group), and 14 (n = 10 for each group) after the first dosing. Cumulative food intake of same mouse as ones in Fig 3E is indicated at each time point which was nearest in sacrifice of animals. (B) Body weight in the siNON- and siINHBE-treated groups on days 2 (n = 6 for each group), 7 (n = 6 for each group), and 14 (n = 10 for each group) after the first dosing. Body weight of same mouse as ones in Fig 3E is obtained at point in time nearest to the time when the animals were sacrificed and at time point before first dosing (Day0). Data are expressed as mean ± SEM. Differences between the two groups were assessed by unpaired *t*-test. **: *P* < 0.01.

The effect of siINHBE on the whole-body metabolic phenotype *in vivo* was examined. Cumulative food intake after the first dosing tended to be lower in the group administered siINHBE (Fig 4A). No difference was observed in water intake between the two groups (data not shown), suggesting that the siRNA treatment had no toxic effects. The db/db mice treated with siNON gained weight, whereas those treated with siINHBE did not. As a result, there was a significant difference in body weight between the two groups on day 14 after the first dosing (Fig 4B).

**Fig 5:**
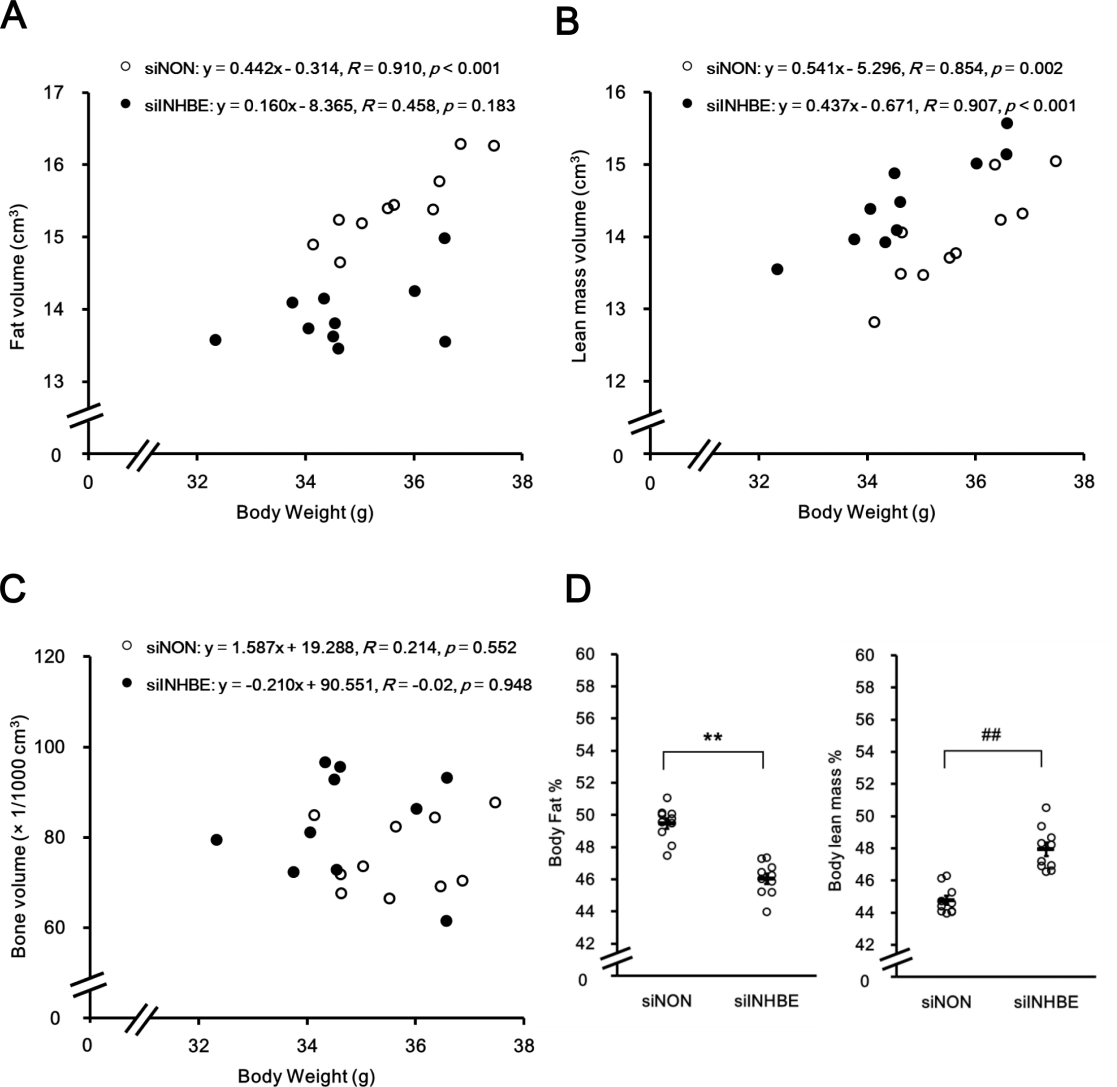
Knock down of hepatic *Inhbe* mRNA in db/db mice altered body composition. Body fat, lean mass, and bone volumes were measured by CT analysis in the siNON- (n = 10) and siINHBE-treated groups (n = 10) on day 10 or 11 after the first dosing. Body fat volume (A), body lean mass volume (B), and bone volume (C) values for each animal were plotted versus body weight values. Body fat volume (A) and body lean mass volume (B) in the siINHBE- versus siNON-treated group were significantly decreased and increased, respectively, by analysis of covariance (*P* < 0.01). (D) Body fat and lean mass percentages were calculated by dividing body fat volume and body lean mass volume by total volume, respectively. Data are expressed as mean ± SEM of 10 animals. Difference between the two groups in body fat percentages in (D) was assessed by unpaired *t*-test. Difference between the two groups in body lean mass percentages in (D) was assessed by Mann-Whitney U test (D). **: *P* < 0.01 (for body fat percentages), ^##^: *P* < 0.01 (for body lean mass percentages).

To examine the effect of siINHBE on body composition, microCT analysis was performed. Fat volume (Fig 5A), lean mass volume (Fig 5B), and bone volume (Fig 5C) were analyzed, and body fat and lean mass percentages were calculated (Fig 5D). siINHBE treatment increased lean mass composition and decreased fat mass volume compared with those of siNON treatment.

**Fig 6:**
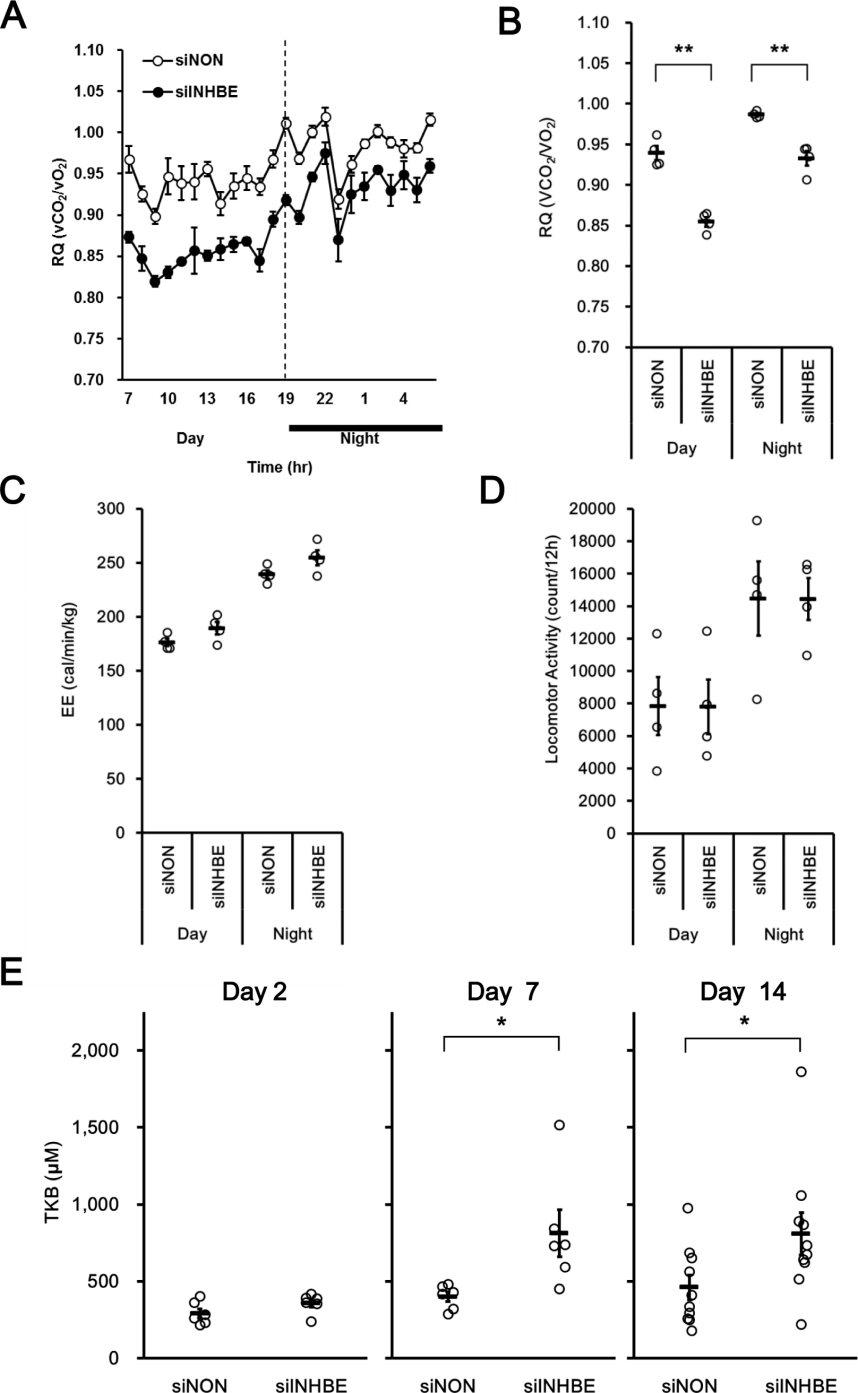
Knock down of hepatic *Inhbe* mRNA in db/db mice caused a switch from carbohydrates to fats as the respiratory substrate and increased fat metabolism. (A) Average energy daytime and nighttime respiratory quotients (RQ) on day 8 after the first dosing in the siNON- (n = 4) and siINHBE-treated groups (n = 4). (B) RQ values over a 24-h period on day 8 after the first dosing in the siNON- (n = 4) and siINHBE-treated groups (n = 4). (C) Average energy expenditure (EE) in the siNON- (n = 4) and siINHBE-treated groups (n = 4) during the daytime and nighttime periods of day 8 after the first dosing. (D) Average locomotor activity in the siNON- (n = 4) and siINHBE-treated groups (n = 4) during the daytime and nighttime periods of day 8 after the first dosing. (E) Plasma total ketone bodies (TKB) on days 2 (n = 6 for each group), 7 (n = 6 for each group), and 14 (n = 10 for each group) after the first dosing. Data are expressed as mean ± SEM. Differences between the two groups were assessed by unpaired *t*-test. **: *P* < 0.01, *: *P* < 0.05.

To explore the mechanism underlying decreased body fat mass composition, an indirect colorimetric study was performed. We observed decreased RQ values following siINHBE treatment (Fig 6A and 6B) without changes in EE (Fig 6C) or locomotor activity (Fig 6D) compared with those following siNON treatment. These results suggest increased whole-body fat utilization as a respiratory substrate by *Inhbe* gene silencing in the liver. In concert with decreased RQ, increased total ketone body concentrations in blood from animals treated with siINHBE were observed compared with those in animals treated with siNON (Fig 6E). These results suggest that the reduced fat volume composition by siINHBE treatment was caused by increased fat utilization.

## Discussion

In the present study, our comprehensive gene expression analysis in humans was comparatively unique and had three novel and superior aspects described below. First, candidate genes were selected by fold change between the Control and Pre-Diabetes groups based on the signal intensity of the probes for each gene in the DNA chip analysis. Selecting candidate genes for each stratification based on the signal intensity for each probe addressed the problem that potentially significant genes with larger signal intensities would not be selected because of smaller degrees of variation of fold changes. Second, when candidate genes were selected by correlation coefficients of HOMA-IR or lipid parameters against the signal intensity for each probe, rank order correlation was utilized to avoid the effect of outliers on the coefficient. Third, when HOMA-IR correlation coefficients were used to select candidate genes that, when suppressed, improved insulin resistance, lipid parameters were also considered to select genes that, when suppressed, improved lipid abnormalities or maintained lipid metabolism in the whole body or liver. Moreover, we preferentially selected genes annotated as secretory proteins to identify novel hepatokines. Three candidate genes were selected through these analyses in humans. Analysis of the three candidate genes in mice using an *in vivo* siRNA technique revealed that *Inhbe* is a possible gene that affects body composition and body weight under obese insulin-resistant conditions. These factors indicated that our analysis in humans was useful and effective to identify genes affecting the whole-body metabolic phenotype.

Our study revealed that hepatic expression of *INHBE* mRNA is upregulated in humans with insulin resistance and obesity. Furthermore, we observed the upregulation of hepatic *Inhbe* mRNA expression in an animal model of obese insulin resistance. Our preliminary functioning screening experiments using siRNA-mediated knockdown of *Inhbe* suggest previously unrecognized roles of *INHBE* in fat metabolism, body weight gain, and body composition. Knockdown of *Inhbe* in obese diabetic db/db mice resulted in reduced weight and fat mass with reduced RQ, increased ketone bodies, and increased body lean mass. These findings suggest that *Inhbe* is causal for sarcopenic obesity via reduced fat utilization.

*INHBE* is a member of the TGF-β superfamily and thought to be a secretory protein in the liver [11, 13]. It has been reported that *INHBE* is predominantly expressed in the liver [13, 15]. And public data regarding tissue distribution of *INHBE* in humans and mice indicates that *INHBE* mRNA expression is very nearly limited to the liver and that its expressions in adipose tissue, skeletal muscle, and brain is much less than in the liver (https://www.ncbi.nlm.nih.gov/gene/83729/?report=expressionhttps://www.ncbi.nlm.nih.gov/gene/83729/?report=expression [in human], https://www.ncbi.nlm.nih.gov/gene/16326/?report=expressionhttps://www.ncbi.nlm.nih.gov/gene/16326/?report=expression [in mice]). Moreover, *Inhbe* mRNA was not detected in the skeletal muscles and epididymal adipose tissue of db/db mouse. Previous studies in animals and *in vitro* have indicated that *INHBE* may be involved in the local regulation of liver cell growth and differentiation [16, 17, 20], growth of pancreatic exocrine cells [21], aggressive behavior [22], and glucose metabolism [18, 23]. Our studies in human subjects indicated that *INHBE* mRNA expression was upregulated under insulin-resistant and obese conditions in subjects with pre-diabetes. This finding is consistent with the results of a previous study indicating that *Inhbe* mRNA was upregulated in the liver of diet-induced obese mice in the fed state [18]. Nevertheless, to our knowledge, this study is the first to report the metabolic significance of *INHBE* expression in human subjects with insulin resistance.

A previous report also indicated that treatment with insulin increased *INHBE* mRNA expression in the hepatoma cell line *in vitro* [18], which is consistent with the results of our study using primary hepatocytes. Thus, hyperinsulinemia under insulin-resistant conditions in humans and animals may be one reason for the increased *INHBE* gene expression in the liver under pre-diabetic conditions. The increased *INHBE* gene expression by insulin despite insulin resistance could be explained by selective insulin resistance [24–26]. Under physiological conditions, insulin receptor signaling pathways in the liver would act to suppress gluconeogenesis and promote lipogenesis. Conversely, in subjects with obesity and type 2 diabetes, impaired suppression of gluconeogenesis and promoted hepatic lipogenesis coexist. Previous reports have demonstrated the contribution of selective insulin resistance to this situation in obese patients with type 2 diabetes [27, 28]. Therefore, the mechanisms of increased *INHBE* expression by insulin under insulin-resistant conditions may be similar to the mechanisms of enhanced lipogenic gene expression in hyperinsulinemia under insulin-resistant conditions. It has been reported that one of the key genes for insulin-induced lipogenesis under insulin-resistant conditions is SREBP-1c [24, 27]. Separately, a previous study indicated that CCAAT/enhancer-binding proteins (C/EBPs) play a key role in the effect of insulin on *INHBE* expression [18]. Crosstalk between SREBP-1c and C/EBPs might act as a mechanism for the promotion of *INHBE* expression by insulin. Further research for clarifying the regulatory mechanisms of promoter activity of *INHBE* is required to test our hypothesis. Also, molecular mechanisms underlying the crosstalk between SREBP-1c and C/EBPs should be investigated.

Previous studies investigating targeted disruption of the *INHBE* gene in mice showed no obvious phenotype with respect to liver growth, differentiation, or regeneration [29], whereas Hashimoto and Funaba [23] reported in their review paper that transgenic mice overexpressing human *INHBE* showed higher insulin sensitivity and lower blood glucose levels than those of control mice. However, the experimental data were not presented, and the role of the *INHBE* gene in insulin resistance and T2D was not discussed. In the present study, knockdown of *Inhbe* mRNA in the liver of db/db mice suppressed body weight gain, increased fat utilization, and decreased body fat composition. Therefore, to our knowledge, this is the first report demonstrating role of *INHBE* in weight gain and fat metabolism.

A positive correlation between HOMA-IR and *INHBE* mRNA expression was observed in human subjects. Moreover, a positive correlation with body mass index was also observed in human subjects. Furthermore, siINHBE suppressed body-weight gain, increased body lean mass, and decreased body fat in the obese animal model. Generally, it is thought that suppressed body weight and decreased body fat mass contribute to better insulin sensitivity and glucose tolerance under obese insulin-resistant conditions. However, glucose metabolism assessed by oral glucose tolerance test was not affected in the present study (data not shown). Metabolic phenotypes observed in patients with growth hormone replacement therapy for growth hormone deficiency may explain the phonotypes observed in our study [30, 31]. Specifically, reduced *Inhbe* mRNA by siRNA might induce a growth hormone-like effect, suppressing protein catabolism in the skeletal muscle and promoting the catabolism of fat in adipose tissue. This is then followed by induction of whole-body glucose intolerance through respiratory substrate switching based on the glucose-fatty acid cycle, as described previously [31]. It might be possible that long-term knockdown of *Inhbe* exerts beneficial effects on glucose metabolism.

Based on our findings, we speculate that the adipose tissue and skeletal muscle are possible target tissues of hepatokine inhibin βE in a form of activin E, a homodimer complex of inhibin βE subunit, via a comparable mechanism of the TGF-β ligand family based on the following findings reported: 1) Recombinant activin B, a homodimer complex of inhibin βB subunit, decreases lipolysis and increases intracellular triglyceride content in 3T3-L1 adipocytes via down-regulation of the expression of adipose triglyceride lipase and hormone sensitive lipase [32]. Likewise, hepatic activin E also potentially plays a significant role in decreasing lipolysis and increasing fat accumulation in adipocytes, because siRNA-mediated knockdown of hepatic *Inhbe* expression in our study resulted in enhanced total body fat utilization together with reduced fat mass. 2) It has been reported that activin A, a homodimer complex of inhibin βA subunit, induces muscle atrophy [33, 34]. 3) Myostatin, a member of TGF-β ligand family, functions as a negative regulator of muscle mass [35–37].

The limitations of the present study include preliminary functional analyses for *Inhbe*. Observed phenotypes were significant but weak and could not be maintained long term in animals treated with siRNA. Thus, it would be difficult to clarify the detailed mechanisms underlying the metabolic function of *Inhbe* in this model. Furthermore, there was a discrepancy between mRNA and protein levels of Inhbe in the siRNA-mediated knockdown experiment; siINHBE suppressed *Inhbe* mRNA levels by ~50% whereas it did its protein levels only moderately. Because only one ELISA system is currently available, we can’t confirm the protein levels by using independent assay systems. However, it may be possible that protein levels of Inhbe are also substantially reduced in mice treated with siINHBE because the phenotypes in our siRNA experiments are consistent with the previous findings in activin B, other family member of TGF-β ligand family as described above [32]. Further studies are needed to confirm our hypothesis regarding function of *Inhbe* by using potent and stable models of *Inhbe* deficiency, such as conditional knockout mice.

In conclusion, the comprehensive gene expression analyses in liver biopsy samples from insulin-resistant people has identified *INHBE* as a candidate hepatokine that alters whole-body energy metabolism under obese insulin-resistant conditions. Our preliminary *in vivo* siRNA study analyzing whole-body metabolic function suggest the possibility that the hepatokine *INHBE* decreases fat utilization and increases fat mass in the mice model of obesity. Therefore, our approach using comprehensive gene expression analyses may be useful to identify functional hepatokines involved in pathophysiology of obesity and T2D.

## Supporting information

S1 Fig Hepatic *Inhbe* mRNA expression in insulin resistant mouse model. Hepatic *Inhbe* mRNA levels were assessed by qRT-PCR in control animals (C57BL/6J mice; n = 6) and an animal model of insulin resistant at 8–12 weeks of age (KK-Ay mice; n = 6). Animals in a freely fed state were sacrificed in the daytime. Data are expressed as mean ± SEM of six animals.
(TIF)

S2 Fig Effect of siRNA for *Inhbe* (siINHBE) on *Inhbe* mRNA expression of mouse primary hepatocytes. Effect of non-targeting siRNA (siNON) and siINHBE on *Inhbe* mRNA expression was examined in mouse primary hepatocytes. Expression levels at each siRNA concentration are indicated relative to expression in medium containing only the transfection reagent. Data are expressed as mean of values from two wells.
(TIF)
