# Supplementary figures and images for: Inhibin βE (INHBE) is a possible insulin resistance-associated hepatokine identified by comprehensive gene expression analysis in human liver biopsy samples
Source: PLoS One. 2018 Mar 29;13(3):e0194798. doi: 10.1371/journal.pone.0194798 (PMC5875797; doi:10.1371/journal.pone.0194798)

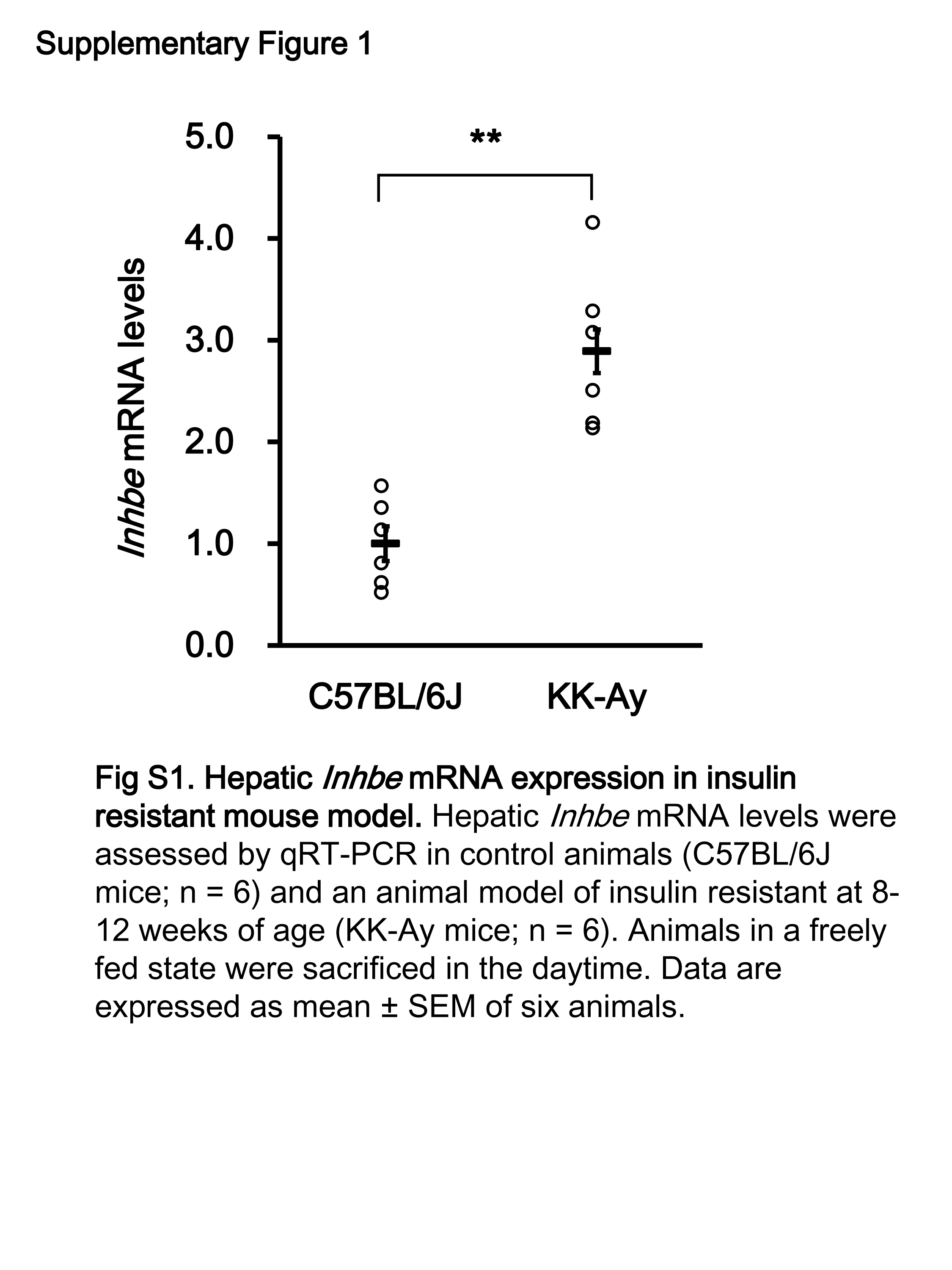

Supplement: S1 Fig — Hepatic Inhbe mRNA levels were assessed by qRT-PCR in control animals (C57BL/6J mice; n = 6) and an animal model of insulin resistant at 8–12 weeks of age (KK-Ay mice; n = 6). Animals in a freely fed state were sacrificed in the daytime. Data are expressed as mean ± SEM of six animals. (TIF) [file pone.0194798.s001.tif]

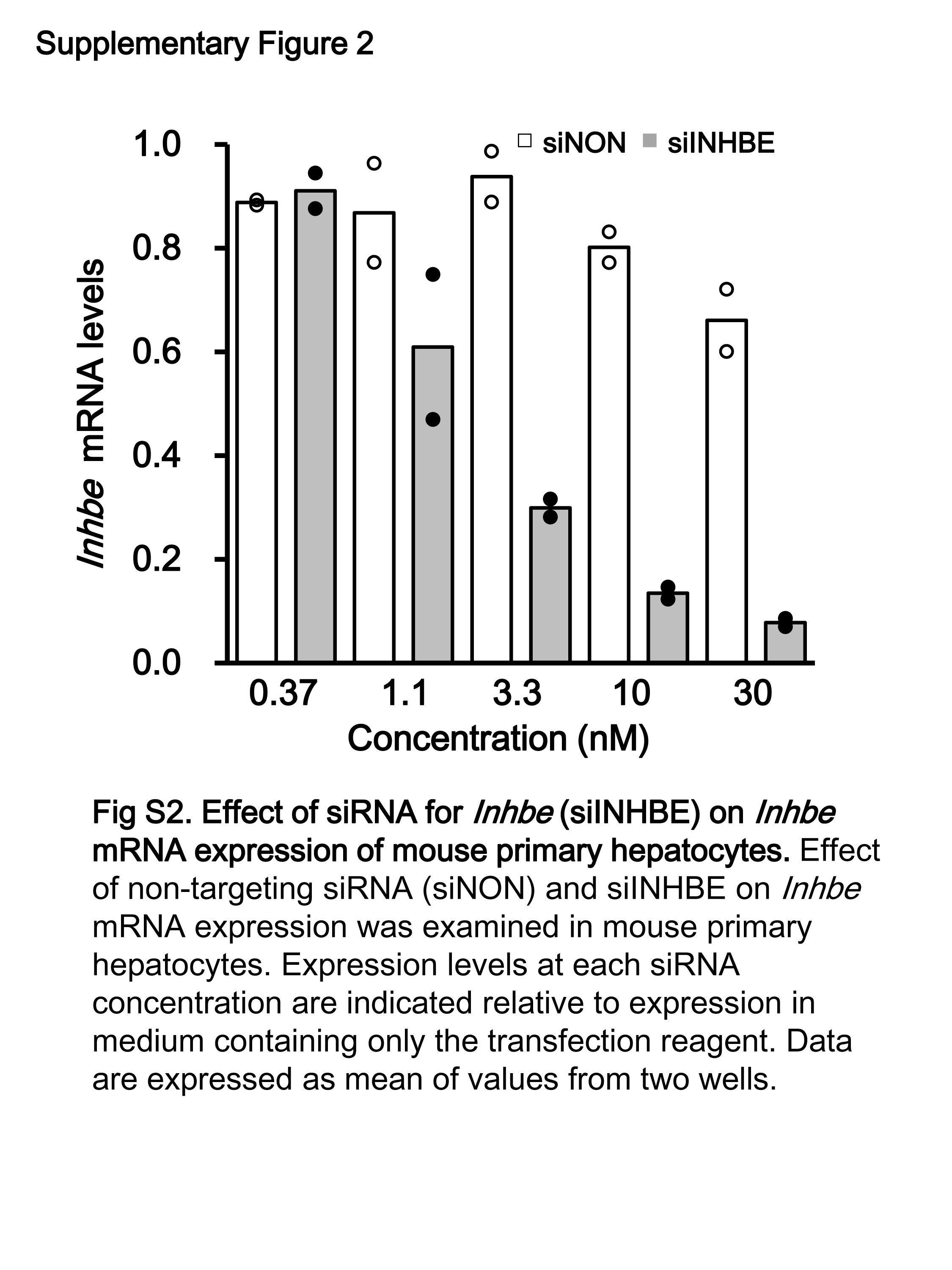

Supplement: S2 Fig — Effect of non-targeting siRNA (siNON) and siINHBE on Inhbe mRNA expression was examined in mouse primary hepatocytes. Expression levels at each siRNA concentration are indicated relative to expression in medium containing only the transfection reagent. Data are expressed as mean of values from two wells. (TIF) [file pone.0194798.s002.tif]
